# “I can’t bend it and it hurts like mad”: direct observation of gout consultations in routine primary health care

**DOI:** 10.1186/s12875-017-0662-9

**Published:** 2017-10-18

**Authors:** Anthony Dowell, Caroline Morris, Lindsay Macdonald, Maria Stubbe

**Affiliations:** Department Primary Health Care & General Practice, University of Otago, 23a Mein Street, Wellington, New Zealand

**Keywords:** Communication, Consultation, General practice, Gout, Primary health care, Qualitative research

## Abstract

**Background:**

Gout is the most common form of inflammatory arthritis and is associated with considerable co-morbidity. It is usually managed in the primary care setting with a combination of lifestyle modification and pharmacological therapy. This study describes patterns of communication about gout observed in interactions between patients and primary care practitioners during routine consultations.

**Methods:**

Secondary analysis of video-recordings of individual healthcare consultations between patients and a range of primary care practitioners (including general practitioners, practice nurses, podiatrists and dietitians) from an archived database. Consultations that included any discussion about gout were eligible for inclusion (*n* = 31) and were not restricted to those where gout was the main presenting complaint. The consultation transcripts were analysed using a qualitative inductive approach from clinical and linguistic perspectives and supplemented with visual observation of the interactions.

**Results:**

Two main themes emerged from the data; the importance of gout and ‘telling’ versus ‘listening’ in consultations. The first theme had two distinct strands; gout as an incidental part of the consultation and the impact of gout on patients. A trend towards more didactic practitioner communication encompassed by the second theme occurred at many different consultation points including diagnosis, in more general post-diagnosis discussion, and when discussing biochemical test results and lifestyle advice. In contrast, when discussion about treatment with medicines occurred a tendency towards a greater degree of listening to patients was observed.

**Conclusion:**

Our observation of the communication patterns in these consultations illustrates an inherent complexity of gout consultations in primary care. Gout may be more important to patients than is often apparent to practitioners in routine consultations. Consultation management needs to take into account the impact of the condition and the balance of information provided around lifestyle advice versus long-term management with medicines.

## Background

Gout is recognised as the most common form of inflammatory arthritis and occurs as a response to the deposition of monosodium urate crystals in the joints [1]. It is associated with a range of co-morbidities including diabetes, hypertension and obesity; it is a chronic condition manifested by acute flares and chronic monosodium urate crystal deposition with differing management options; [1] it is usually managed in primary care. Effective management is by advocating a combination of lifestyle modification and pharmacological therapy [1, 2]. In contrast to some other chronic conditions (e.g. asthma), a clearly defined target value for a biochemical test result (serum urate level ≤ 0.36 mmol/L) is associated with better outcomes for patients whose symptoms recur [1, 2].

Challenges to effective primary care management include doctors’ and patients’ perceptions of the condition and the need to address gout and its co-morbidities within short consultation time-frames [3]. In line with primary care consultations for other conditions, gout may be the patient’s main presenting complaint or surface incidentally during the consultation, and be raised by either the patient or their doctor [4, 5].

A number of primary care based qualitative [6–14] and quantitative [15–17] studies have investigated patient and/or practitioner experiences and perceptions of gout. Evidence shows that the condition adversely affects sufferers’ lives in a substantive way. Gout is the cause of considerable pain and has a negative impact on patients’ home, work and social lives [6–8, 10–13]. Furthermore, the diagnosis can be a source of stigma for patients resulting from the stereotypes that exist around the condition’s perceived link to lifestyle and alcohol use [10–13].

Although doctors are aware that a gout diagnosis is a potential source of stigma for patients, [12] feelings of being stigmatised may contribute to sub-optimal management, [18] alongside a lack of patient [6–8, 12, 13, 19] or practitioner [12, 14, 18] knowledge about the appropriate management of the condition.

Patients and practitioners often have differing views about gout and the ease with which it can be managed, [9] with management perceived by doctors as being further complicated by the presence of patient co-morbidities. [12, 14] Although treatment guidelines exist, [20–22] they are not always applied by clinicians in practice [18, 19].

Some patients are aware of specific food or drinks precipitating a personal acute gout attack, [12] while others show a lack of awareness that lifestyle modification may be one way of managing the condition, [19] and poor awareness of common food triggers [16, 23]. Although modifying lifestyle and diet is a recognised part of gout management [1, 2] and recommendations encompassing these areas form part of national guidelines for gout management, [20, 21] the evidence supporting their inclusion is low in the evidence hierarchy. More recently the question of the balance of information provision to patients about lifestyle measures versus management with medicines has been raised as an important issue to address [11, 14].

There is a significant body of research dating back to the 1970s involving the direct observation of patient-health professional communication [24]. In the past two decades interactional sociolinguistics and conversation analysis methodologies have been used to unpack the “background orientations, individual experiences, sensibilities, understandings, and objectives that inhabit the medical visit” [25]. Previous work demonstrates the ways in which doctors and patients often pursue quite different agendas in the history-taking phase of the consultation. While pursuing their biomedical agenda, doctors may suppress patients’ expressions of their “lifeworld” concerns and circumstances even though these often had a real bearing on their medical problems and outcomes [26].

Previous qualitative research with gout patients and the primary care practitioners involved in their care has taken place outside of the consultation setting thus relying on participant recall of the events that took place. To date, no previous study has explored directly observed interactions between patients with gout and the practitioners who care for them during routine consultations. This study aims to provide an initial description of the language and patterns of communication relating to gout observed in interactions between these parties within the day to day context of primary care consultations. A clearer understanding of the consultation process has the potential to help identify ways of improving patient care.

## Methods

### Data source

Consultation data are sourced from the Applied Research on Communication in Health (ARCH) Corpus at the University of Otago, Wellington, New Zealand (see Table 1). The collection of all Corpus data and guidelines for subsequent use have been approved by the national New Zealand Health and Disability Ethics Committee [27].

**Table 1:** Background information about the ARCH Corpus database [27]

This Corpus houses a digitally stored collection of patient / practitioner consultation data that includes video-recorded consultations and verbatim transcripts.
Included data has been collected since 2003 as part of funded studies.
These studies include the:
- Interaction Study (IS) exploring clinical decision-making when rationing is explicit;
- Tracking Study (TS) exploring communication processes throughout a single complete episode of care of patients referred from primary to secondary care;
- Diabetes Study (DS) tracking the contact of newly diagnosed patients with Type 2 diabetes with healthcare professionals over a six-month period.
The combined dataset for the IS and TS comprised an unselected sample of 183 routine consultations involving 15 general practitioners recorded in 21 participating general practices in the Wellington region of New Zealand between 2004 and 2007. The data for the DS was purposively collected in Wellington and Auckland; for this study, 36 patients newly diagnosed with type 2 diabetes were recruited prospectively via 21 general practices (6 of which had participated in the previous studies) and tracked for a period of 6 months between 2008 and 2011. Their consultations with general practitioners and nurses at the general practice and related consultations with allied health professional staff were video-recorded.
All consultations were made in the course of ‘practice as usual’ and are therefore typical of routine interactions occurring in New Zealand healthcare.
Written consent was sought from participants in these original studies to use their data for secondary analysis.
At the time of sampling the ARCH Corpus included 418 video-recorded consultations recorded between 2003 and 2011, 337 of which were eligible for inclusion. These comprised 247 individual patients, 30 general practitioners, 31 nurses and 15 other practitioners.

### Identification of gout consultations

The ARCH Corpus includes a Microsoft Access database populated with full demographic information about every participant, research site information and free text content logs of each consultation. The logs are prepared by a research nurse according to a standard template and include information about the main topics discussed, outcomes of the consultation (including prescriptions and referrals), and a minute by minute summary of key events and content. The logs thus capture any complaint or topic mentioned incidentally in a consultation, in addition to the main presenting complaint(s). The database and logs link electronically to full verbatim transcripts and the original audio and video-recordings to facilitate subsequent more detailed analysis, but the latter cannot be queried directly via the database.

A query was run on the Microsoft Access database using the keyword “gout”. It is theoretically possible that not all potentially relevant consultations in the Corpus were identified, but this is unlikely as gout is a readily identifiable topic of discussion. A subsequent cross-check using the names of common gout medicines as search terms did not yield any additional consultations. The purpose was the construction of a relevant dataset adequate for the purpose of undertaking a descriptive qualitative analysis, not to investigate frequency of occurrence.

The term gout was present in the logs of 31 individual patient consultations with primary care practitioners. The aim was not specifically to identify consultations where gout is the main presenting complaint, but rather to observe how gout is addressed in the context of routine consultations. Twenty of the consultations analysed originated from the DS (see Table 1), and the majority of those contained elements of a more structured consultation format based on a national protocol for diabetes care. The remaining 11 general practitioner (GP) consultations were derived from the IS study (*n* = 5) and the TS study (*n* = 6) respectively. Brief demographic characteristics of the patients and practitioners included in these consultations are shown in Table 2.

**Table 2:** Demographic characteristics of patients and practitioners involved in the gout consultations (*n* = 31)

*Patient characteristics*	
• 18 individual patients	
- Male *n* = 13; female *n* = 5	
- Age: mean 54 years; median 52 years	
- Age range: 36 to 67 years	
*Health practitioner characteristics*	
• 17 individual practitioners	
- General practitioners *n* = 10	
- Nurses *n* = 4	
- Podiatrists *n* = 2,	
- Dietitian *n* = 1	

### Data analysis

This study was based on the verbatim transcripts and video-recordings of interactions between patients and practitioners. Themes were derived iteratively using a qualitative inductive approach, [28–30] following the process shown in Table 3; our overarching aim being to report on the emerging range of issues and communication styles without pre-conceived assumptions.

**Table 3:** Data analysis process

1. CM identified and coded conversation sequences related to gout in the transcripts. CM then reviewed the video-recordings to confirm and enrich the analysis. A note was made of any important contextual non-verbal communication from either party e.g. nodding of the head, smiling, sighing, listening attentively, examining the patient, turning away from the patient.
2. Initial coding included:
- how gout was introduced into a consultation and by whom
- the location within the consultation
- the importance of the condition from the patient perspective
- diagnosis and treatment of the condition
- emphasis placed on biochemical test results and dietary and lifestyle advice
3. Themes and sub-themes were identified from the data. Initial interpretations of the clinical relevance and importance of the themes derived were debated and discussed between CM, a pharmacist and experienced qualitative researcher, and AD, the general practitioner member of the project team.
4. LM and MS, researchers with experience in linguistics, contributed to a second round of discussion and interpretation. Examples of refinements at this point include:
- “Gout as an incidental part of the consultation” and “Impact of gout for patients” being combined as sub-themes of the over-arching theme “The importance of gout”
- A linguistic interpretative viewpoint to the differences observed around lifestyle and medicines
- Acknowledgement of the importance and possible ambiguity of ‘semi-verbal’ communication (e.g. the use of “mm” by participants)
5. Disagreements were resolved by consensus.
6. All authors reviewed and agreed the final themes and interpretation

## Results

Two key and important themes that emerged from our data relating to the importance of gout and telling versus listening in consultations are the focus of this paper and described in more detail below. These, together with other themes identified from the dataset are shown Table 4.

**Table 4:** Themes derived from the data

Themes that are the focus of this paper	
• The importance of gout	
- Gout as an ‘incidental’ part of the consultation	
- The impact of gout on patients	
• ‘Telling’ versus ‘listening’ in consultations	
Medicine-related themes^a^	
• Level of patient knowledge	
• Patient attitudes to medicines	
• Attributes of practitioner communication	
- Delivery and content of information provided	
- Taking the opportunities presented	
Other themes	
• Patient interpretation of symptoms	
• Patient understanding of gout management	
• Patient understanding of uric acid levels	

^a^Described in detail elsewhere [40]

### The importance of gout

To aid interpretation of the results contextual background information about the 31 gout consultations is shown in Table 5.

**Table 5:** Contextual information about 18 individual patient’s gout consultations (n = 31)

Consultation Identifier*	Patient age (years)	Practitioner	Incidental or presenting complaint (PC)	Initiator of gout conversation	Point of consultation gout first mentioned	Discussion length; context	Gout medicines mentioned	Lifestyle advice/diet mentioned	Uric acid levels mentioned
DS-GP01–04	41–50	GP	Incidental	Patient	Early	Short; no current symptoms	Yes	Yes	Yes
DS-NS10–01	41–50	Nurse	Incidental	Practitioner	Late	In passing^#^	No	Yes	Yes
DS-NS14-02a	51–60	Nurse	Incidental	Practitioner	Late	In passing^#^	No	Yes	No
DS-GP19-02b	51–60	GP	Incidental	Patient	Start	Substantive; no current symptoms	Yes	Yes	Yes
DS-NS14-02b	51–60	Nurse	Incidental	Patient	Start	Short; No current symptoms	Yes	Yes	No
DS-GP29–01	41–50	GP	Incidental	Patient	Start	Substantive; acute flare, new diagnosis	Yes	Yes	Yes
DS-NS13-01a	31–40	Nurse	Incidental	Patient	Middle	Short; patient thought they had gout	No	Yes	Yes
DS-NS13-01b	31–40	Nurse	Incidental	Practitioner	Early	Short; following up discussion of previous visit where patient thought they had gout	Yes	No	No
DS-GP18–01	31–40	GP	Incidental	Practitioner	Middle	Substantive; discussion of preventive therapy	Yes	No	No
DS-HP06-01a	31–40	Dietitian	Incidental	Patient	Start	Short; struggling with gout	Yes	Yes	No
DS-HP07-01a	31–40	Podiatrist	Incidental	Patient	Start	Substantive; no current symptoms	Yes	Yes	No
DS-NS13-01c_GP18	31–40	Nurse & GP	Incidental	Patient	Start	Substantive; acute flare	Yes	No	No
DS-HP06-01b	31–40	Dietitian	Incidental	Practitioner	Start	Substantive; acute flare	Yes	Yes	No
DS-HP07-01b	31–40	Podiatrist	Incidental	Practitioner	Start	Substantive; no current symptoms	Yes	No	No
DS-NS13-01d_GP18	31–40	Nurse & GP	Incidental	Patient	Early	Substantive; acute flare subsiding	Yes	No	Yes
DS-NS13-04d_GP21	61–70	Nurse & GP	Incidental	Patient	Start	Short; mention of recent flare	No	No	Yes
DS-NS25–01	51–60	Nurse	Incidental	Practitioner	Early	In passing^#^	Yes	No	No
DS-NS27–02	41–50	Nurse	Incidental	Practitioner	Close	In passing^#^	Yes	No	Yes
DS-HP10-02a	41–50	Podiatrist	Incidental	Patient	Middle	Short; making conversation	Yes	No	No
DS-HP10-02b	41–50	Podiatrist	Incidental	Patient	Middle	Short; making conversation	Yes	Yes	Yes
IS-GP02–03	41–50	GP	Incidental	Practitioner	Start	Substantive; no current symptoms	Yes	No	No
IS-GP02–14	41–50	GP	PC	Patient	Start	Substantive; acute flare, pre-existing diagnosis	Yes	No	Yes
IS-GP03–06	61–70	GP	Incidental	Practitioner	Late	In passing^#^	Yes	No	Yes
IS-GP07–05	61–70	GP	Incidental	Practitioner	Start	In passing^#^	Yes	No	No
IS-GP07–06	41–50	GP	Incidental	Practitioner	Middle	In passing^#^	Yes	No	No
TS-GP03–08	51–60	GP	Incidental	Practitioner	Middle	In passing^#^	Yes	No	No
TS-GP03–12	61–70	GP	Incidental	Practitioner	Start	In passing^#^	Yes	No	No
TS-GP03–17	61–70	GP	Incidental	Practitioner	Middle	Short; gout considered before being eliminated	Yes	No	No
TS-GP08–10	51–60	GP	Incidental	Practitioner	Early	In passing^#^	Yes	No	Yes
TS-GP09–05	41–50	GP	PC	Patient	Start	Substantive; acute flare, pre-existing diagnosis	Yes	Yes	No
TS-GP14–04	61–70	GP	Incidental	Practitioner	Middle	Substantive; no current symptoms	Yes	No	No

*GP* General practitioner

^*^Consultations prefixed by DS were part of the Diabetes Study, IS the Interaction Study and TS the Tracking Study; a brief description of each study is shown in Table 1

^#^In the context of general patient review / medicines and/or blood test review

The data show that gout is perceived as an important issue by patients and practitioners in two distinct ways. Firstly, gout can ‘intrude’ into the consultation, even when it is not the prime reason for consulting; it is the presenting complaint in only two consultations. Secondly, when patients introduce the condition by name or describe symptoms of gout it becomes clear that it has a significant impact on their well-being. The abbreviations used in the quotations below are as follows: GP = general practitioner; NS = nurse; PT = patient; POD = podiatrist; DIET = dietitian.

#### Gout as an ‘incidental’ part of the consultation

In many consultations conversations about gout stem from the practitioner’s general review of the patient or during a consultation about a different, but related, morbidity e.g. diabetes:

*NS: “If we can check everything [blood tests] … it’s part of the annual review that’s very important … we also check your uric acid, that’s for, you know, your gout” (DS-NS27–02)*

Three patients intentionally and explicitly re-focus their diabetes review visits to gout by raising the issue at the beginning of the consultation. Following a very short discussion about their weight this patient abruptly changes the subject to gout symptoms:

*PT: “Right now … look, [patient shows GP their foot], I can’t touch it, I can’t bend it and it hurts like mad … [I’ve had it] for a week and a half … it hurts” (DS-GP29–01)*

Re-focusing a consultation to gout is not always patient-driven. This GP makes gout a focus right at the start of the consultation despite the patient’s primary reason for visiting being influenza-like symptoms:

*GP: “You saw [Doctor’s name] since I last saw … and he thought you had resistant gout” (IS-GP02–03)*

A lengthy dialogue then ensues around gout and its treatment before the patient sighs heavily and clearly and deliberately re-focuses the consultation to the presenting complaint.

On other occasions gout is merely alluded to in passing; most commonly relating to a previously prescribed medicine:

*“ … and I also gave you some [medicine name] for your gout back then as well” (TS-GP03–12)*

#### The impact of gout on patients

Gout is an important topic of patient concern that may be raised within a consultation with any member of the primary care team (including GPs, nurses, dietitians, podiatrists) in this dataset. The personal impact of the condition and more general aspects of management are all discussed, irrespective of whether the patient is currently experiencing an acute disease flare. Furthermore, counter to an often anecdotally perceived clinical picture of easily managed gout flares restricted to a single anatomical area, patients highlight both the severity and the duration of attacks:

GP consultation:

*PT: “at night it is really swollen and I’ve had to put my foot out of the bed … night-time it is worse … and I’ve been wearing [sandals] because I can’t have anything touch it …” (DS-GP29–01)*

Podiatrist consultation:

*PT: “I had a big wave of gout for a little while [that] lasted for about a good three weeks, it took a while to wear off”*

*POD: oh you poor thing, … whereabouts did it affect you the most?*

*PT: … “it started from my toes, round to my ankle and then it just stayed there for a while and it just slowly headed up this way [points up the leg].” (DS-HP07-01b)*

Patients also comment on the impact of the lifestyle changes they make to help manage their condition. Most patients with diagnosed disease are aware of some of their potential dietary triggers:

*PT: “I used to eat mussels but it gives me a lot of trouble with my gout … I keep away from foods that make me sick … ” (DS-NS14-02a)*

Despite this knowledge, there is, however, a sub-theme of continuing to eat foods which patents knew they should avoid.

*PT: “… I ate all these wrong kind of foods and I gave myself gout which is a real drag cos I haven’t had an attack for ages, but it’s totally self-inflicted … I ate all kinds of shell fish and [expletive] chocolate and all the things that triggered it” (TSGP09–05)*

The ways in which patients in our data typically conceptualised gout and their experience of its symptoms and management are clearly displayed in the language used in these excerpts to describe symptoms and the degree to which discomfort is beyond what is considered normal. As shown in the podiatrist consultation this usually elicits a sympathetic response which serves to validate the severity of the patient’s reported symptoms and potentially demonstrates the justification for medical attention.

### ‘Telling’ versus ‘listening’ in consultations

Despite demonstrating high levels of professional expertise in patient management, practitioners often appeared to find patient-centred approaches to gout communication challenging. In this dataset practitioners tended to employ a more didactic style of communication by ‘telling’ rather than listening, often framing information in a biomedical way that is likely to be out of step with the patient’s ‘lived’ experience.

The ‘telling’ consultation style can be observed at the beginning of a patient’s gout ‘career’ as shown in the following consultation where gout is discussed for the first time. The GP comprehensively examines the foot while eliciting information about symptoms and the limits they place on the patient’s ability to function. The patient maintains eye contact and listens attentively as the GP explains the aetiology of the condition in a biomedical way, identifying uric acid as the cause, but linking back to the symptoms the patient is experiencing:

*GP: “We call it gout … there is one acid called uric acid … if the level in the blood is high it forms crystals and they deposit in the joint and make the joint painful - it is painful and swells up at times ” (DS-GP29–01)*

Although the patient immediately responds by articulating their difficulty in managing the pain, particularly at night, the GP moves straight into providing information on treatment options and lifestyle changes. The didactic approach means there is little attempt to identify the patient’s understanding of gout or the psychological or social impact of the condition.

This communication style persists in more general gout discussions following diagnosis. Here, the patient volunteers that they take medicines for gout. The podiatrist immediately provides information while looking down at the patient’s feet, without first ascertaining the patient’s knowledge of their condition:

*POD: “The gout tablet allopurinol is to prevent you having a gout attack”*

*PT: The colchicine*

*POD: “That’s for when you get the attack … Gout’s very painful as well, it’s a type of arthritis, it causes intense pain and inflammation in a joint usually in these ones [points to the toes] but I have seen it round the ankles and down the sides of the feet … if they leave it untreated you can get damage to the joints which is permanent damage …” (DS-HP10-02b)*

As uric acid levels are a defined biochemical marker for the diagnosis of gout, GPs often discuss this condition with patients from a biochemical perspective:

*GP: “OK, it looks to me like you’re doing well with the diabetes and the gout. The uric acid level in that last blood test was normal and we probably should get you to have another blood test at some stage so I might just print you [off a blood test form].” (DS-NS13-01d_GP18)*

The GP gave information about uric acid levels without any discussion about how the patient currently felt about their gout or its impact on them.

In this cohort patients often appeared to have little understanding of the importance and meaning of uric acid levels, although one did raise the issue in a routine diabetes check when the nurse is discussing their diet:

*PT: “… [I’m] cutting down [on certain foods], because of my cholesterol and my uric acid …”*

This immediately precipitates a short conversation that focuses on information provision:

*NS: “Do you suffer from gout? Yeah, and you’ve had good information about things that trigger your gout? Do you go on the internet at all?”*

*PT: now and then*

*NS: “OK, so I’m just going to write up your care plan and we’re really nearly there” (DS-NS10-01)*

Conversations around dietary triggers for gout are a feature of many consultations spanning all practitioner groups. In the following example the GP appears to assume that the patient already has dietary information, rather than taking the opportunity to elicit the patient’s understanding of triggers for gout and what it means to them personally. The patient’s reply precipitates an information giving approach from the GP:

*GP: “… you’ve got information about the foods to avoid with regard to your gout”*

*PT: I don’t have any information about that, but I just think in my mind that I don’t really eat too much red meat, maybe fish, but I haven’t had any information about that*

*GP: “OK well I’ll give you something to read about that, about the sorts of food to avoid [and] what tends to precipitate it; but the things you said are all the things that tend to bring it on and so just try to eat those in moderation or avoid them if they do cause flare-ups. Some of the other main things are shell fish, also alcohol, beer and carbonated drinks as well like fizzy drinks” (DS-GP19-02b)*

At this point the patient volunteers information about their diet which the GP assesses and then reframes biomedically in the context of uric acid levels:

*PT: “I avoid them all except sea food, I ate mussels, is that OK”?*

*GP: Did it cause any symptoms after you had the mussels?*

*PT: No*

*GP: “if you’re having small amounts and you don’t get symptoms that should be OK although your uric acid level in your blood … it is still high” (DS-GP19-02b)*

The patient maintains eye contact throughout the conversation and nods his head indicating an unspoken acknowledgment of what has been said.

Similar conversations with other practitioners are also framed in a biomedical way, with a focus remaining on ‘telling’ the patient rather than attempting to gain an understanding of their perspective. In this podiatrist consultation the patient mentions a pamphlet about diet precipitating this response:

*POD: “I think it’s about the only type of arthritis that it’s been proven that certain food will have an effect on it and you’re supposed to avoid foods that are high in uric acid I think like shell fish … and alcohol.” (DS-HP10-02b)*

Medicines play a key role in the optimal management of gout. In this topic area the style of consultation was observed to move away from a didactic one towards a greater degree of listening to the patient. In the following example the GP negotiates options and checks that the patient is happy with a decision before it is finalised:

*GP: “we should be probably giving you something to prevent the gout”*

*PT: oh OK, cos I do get it quite regularly especially in my ankle and my toe and it just throbs and I know that runs in my family*

*GP: we’d start you with a low [dose] - you can choose to start the tablet now or we can put it off for a while, it’s up to you really …*

*PT: just do it, just get it over and done with*

*GP: “[so], we start with a low dose and sometimes it can flare it up a little bit so it’s probably good to do it now if you haven’t got any gout … it’s probably better to just get this a little bit sorted out for you if we can” (DS-GP18-01)*

Many patients were willing to consider long-term preventive treatment and others were very positive about it. Although there were no examples of patients refusing allopurinol specifically, some patients are less than comfortable with medicines per se:

*PT: “… I don’t like tablets … I don’t want to take any more tablets …” (DS-GP19-02b)*

In this case the GP acknowledges the patient’s view and carefully introduces the possibility that allopurinol may be the best option if the clinical situation alters. The patient non-verbally indicates their acceptance by a nod of their head:

*GP: “… so I think if you do start having regular flare-ups then we should look again at putting you back on allopurinol.” (DS-GP19-02b)*

## Discussion

To our knowledge, this study is the first to directly observe conversations about gout in routine primary care consultations. It provides insights into how the importance of gout is framed by both patients and practitioners in primary care, and the way in which gout stories are elicited and placed in the context of management.

In line with other studies, our results show that gout has a significant impact on patients’ functionality and well-being [6–8, 10–13]. It can be the cause of significant pain as shown by the demeanour of patients as they described their symptoms in the consultation, and can have a substantial impact on their lives. A notable feature is the use of “extreme case formulations” [31] (e.g. “*a* *big wave* *of gout”*, “*I can’t have* *anything* *touch it”, “* *all* *the things that triggered it”*). Phrases such as these, along with the body language at the time of their utterance clearly convey the impact on the patient and also serve to strengthen the legitimacy of their description in the face of possible doubt [31]. Patients also clearly articulated the discomfort they were experiencing and how it persists or varies over time, reinforcing their justification for seeking medical attention [32].

Whether the issue of gout arose as a presenting complaint or as an incidental part of the consultation practitioners clearly listened to patients’ concerns and were sympathetic but in the cases studied moved quickly to a biomedical framing of the condition. Understandably the patient focus is on the pain they are experiencing, while the practitioners’ focus remains on disease management. Irrespective of circumstances, in common with other studies investigating chronic disease, often only limited time was spent attempting to understand the patient’s perspective and the wider impact of the disease. The use of a biomedical model, language and framing heightens the challenge of exploring the psychosocial implications of gout in the consultation [33].

Although patients voiced agendas usually focus on symptoms, [34] doctors may also have an agenda [5]. A similar gap in the conceptualisation of illness has been previously identified in a study of a diverse range of general practice consultations [5] and specifically in diabetes, [33] with patients focusing on physical symptoms while doctors focused on prevention, medicines and tests. In this dataset gout was more often raised as an incidental part of the consultation rather than a presenting complaint, a feature that has also been described in relation to osteoarthritis [4].

GP consultations are known to be complex with many involving several patient problems covering multiple disease areas [5]. It is well known that there is time pressure to adequately consider issues raised incidentally, or beyond the presenting reason for encounter. GPs have previously described areas of differential prioritisation. [4] Influencing factors include patient safety, prioritising problems where they considered they could be most helpful and prioritising areas where financial incentives exist. Issues that were introduced at a later point in the consultation were sometimes perceived as unproblematic for the patient [4]. In the present study gout was rarely the presenting complaint, and the GP conversations illustrate the challenges faced when a condition of concern to the patient is not presented as a high priority within the consultation agenda.

A key finding from this study, clearly identified by the verbal interaction and additional insight gained from viewing the body language, is the different priority given to ‘listening’ and ‘telling’ in gout consultations when discussing lifestyle versus medicines. In this sample there was a more didactic focus on ‘telling’ patients what to do when lifestyle issues such as diet are discussed irrespective of the current status of the patient’s condition. The didactic ‘telling’ style of lifestyle advice has parallels with other studies of directly observed consultations where rather than adjusting information to the specific situation of a patient only generic information about lifestyle behaviour is provided [35]. We have previously noted a similar tendency in diabetes-related lifestyle discussions in the larger set of consultations analysed for the DS, from which the dataset for the current study was drawn [36]. The impact on patients’ lives of recommended lifestyle changes for chronic conditions such as gout can be significant, and a patient’s life circumstances may in turn influence their willingness and ability to accept and follow the advice proffered where this conflicts with the patient’s non-medical priorities [37–39].

Consultation conversations became more measured with a greater focus on listening when the topic is treatment with medicines. With medicines practitioners often asked open questions and took a more patient-centred approach. This feature of our dataset has been described in more detail elsewhere [40]. This difference may arise because treatment with medicines is perceived as potentially more problematic clinically, resulting in the “tentative hedging” we observed.

A gout diagnosis is a recognised source of stigma for patients due to its association with negative lifestyle choices. [8, 10, 11, 13]. A focus on lifestyle by practitioners may amplify this, leading patients to feel that they are being judged [11] and that their condition will not be taken seriously, [18] thereby making them less likely to seek help [14]. Indeed, some primary care practitioners have expressed frustration that patients’ gout symptoms persist because they do not adhere to dietary advice [14]. In addition, other work has identified that some practitioners lack confidence in the treatment of gout, [12] are unfamiliar with treatment guidelines, [12, 18, 19] place a higher priority on lifestyle advice [14] and focus on treating acute attacks underestimating the long-term complications of the disease [18, 19]. A key challenge for practitioners is likely to be finding the optimal balance between the delivery of lifestyle advice and advice around the more effective, but potentially more challenging conversation of treatment with medicines [11].

In this study patients were willing to talk about gout to any available practitioner. It is thus important that primary care practitioners make use of opportunities for a more pro-active approach to gout management, especially as part of a multidisciplinary team. Gout management strategy ‘packages’ (information / education; tight monitoring; reassurance) with more clearly defined practitioner roles have been shown to be effective in specialist hospital clinic practice, and it is possible that elements of this approach might be of use in primary care [41].

A strength of the study is the direct observation of gout discussions in routine consultations. This provides an opportunity to see how gout is made relevant in consultations where it has not been the prime reason for the encounter. We were also able to view interactions between patients and a range of primary care practitioners. This provides information about interactions within the context of the primary care team, aligning with the desired trend towards better integration of the wider team in chronic disease management [42]. A potential limitation of the study is that differences may exist between patients who did not consent to being videoed and therefore not included in the database used for the study and those who were happy to be recorded and therefore included. A further limitation includes the possibility that the presence of the video-recorder may have led participants to behave differently from ‘normal’. However, while all appear aware that the consultation is being recorded at the beginning, this diminishes as the core business of the consultation proceeds [43]. Although this study was conducted within the New Zealand healthcare system, the commonalities of long-term condition management in many high-income countries give the findings international relevance.

## Conclusions

This study illustrates an inherent complexity of gout consultations in primary care. Gout may be more important to patients than is often apparent to practitioners in routine consultations. Consultation management needs to take into account the impact of the condition and the balance of information provided around lifestyle advice versus long-term management with medicines.

## Abbreviations

ARCH: Applied research on communication in health
DIET: Dietitian
DS: Diabetes study
GP: General practitioner
IS: Interaction study
NS: Nurse
POD: Podiatrist
PT: Patient
TS: Tracking study
